# Revision Total Knee Arthroplasty With the Use of Restricted Kinematic Alignment Protocol: Surgical Technique and Initial Results

**DOI:** 10.3389/fsurg.2021.721379

**Published:** 2021-08-26

**Authors:** Lazaros Kostretzis, Gabriel Bouchard Roby, Sagi Martinov, Marc-Olivier Kiss, Janie Barry, Pascal-André Vendittoli

**Affiliations:** ^1^Département de Chirurgie, Hôpital Maisonneuve-Rosemont, Université de Montréal, Montréal, QC, Canada; ^2^Clinique Orthopédique Duval, Laval, QC, Canada; ^3^Personalized Arthroplasty Society, Atlanta, GA, United States

**Keywords:** revision, knee, arthroplasty, technique, patient reported outcome measures, restricted kinematic alignment, mechanical alignment, kinematic alignment

## Abstract

**Purpose:** Kinematic alignment (KA) for primary total knee arthroplasty (TKA) has been shown to provide equivalent or better results to mechanical alignment (MA). The use of KA in revision TKA to restore the individual knee anatomy, kinematics, and soft-tissue balance, has not been documented yet. The purpose of this study is to describe the technique for performing TKA revision using the restricted KA (rKA) protocol and to report (1) rerevision rate and adverse events, (2) patient-reported outcome measures (PROMs), and (3) radiological signs of implant dysfunction related to this technique.

**Methods:** The rKA protocol was used in 43 selected TKA revisions cases suitable for the technique. Adverse events, reoperation, revision, and their causes were recorded. In addition, PROMs assessed by WOMAC score and radiographic evaluation to identify signs of implant dysfunction were documented at last follow-up.

**Results:** After a mean follow-up of 4.0 years (0.9–7.7, ±2), only one rerevision (2.3%) was required for persisting instability (polyethylene liner exchange from posterior stabilized to a semi-constrained). Short-cemented stems were used for both the femur and tibia in 28 (65%) cases, for the femur alone in 13 (30%) cases, and no stems in two cases. In 31 (72%) cases, a standard posterior stabilized tibial insert was used, while 12 (28%) cases required a semi-constrained insert. The mean WOMAC score was 34.4 (0–80, ±21.7). Mean postoperative arithmetic hip-knee-ankle angle (HKA) was 0.8° varus (from 5° varus to 4° valgus), mean mechanical distal femoral angle was 1.7° valgus (from 2° varus to 5° valgus), and mean mechanical tibia proximal angle was 2.2° varus (from 5° varus to 1° valgus). No radiological evidence of aseptic loosening or periprosthetic radiolucencies were identified.

**Conclusion:** Although current revision TKA implants are not ideal for revision TKA performed with rKA, they are an appealing alternative to MA, especially in cases of early, non-wear-related, unsuccessful MA TKAs. rKA TKA revision using short-cemented stems in conjunction with meticulous preoperative planning is safe in the mid-term.

**Level of evidence:** IV

## Introduction

The number of total knee arthroplasties (TKAs) performed worldwide is constantly increasing as the population is growing and indications for TKAs are widened to include younger patients (1). It has been estimated that revision knee surgeries could increase by 601% in the USA (2), and up to 332% in England and Wales (3), by 2030. Revision TKA procedures are highly complex procedures and have inferior survival rates and poorer functional outcomes than primary arthroplasties (4, 5). Currently, the main reasons for revision include infection, loosening, instability, and pain (6). As TKA implants have improved over the years, rates of aseptic loosening have gradually lowered, and it is now the second main reason for revision (7).

Despite the advances in implant technology, patient dissatisfaction has remained relatively high at 15–20% (8). In addition, up to 50% of patients report persisting residual symptoms such as pain, stiffness, and instability (9, 10). Some authors believe that many unsatisfactory outcomes may be due to anatomical changes linked to the mechanical alignment technique (11–13). In primary arthroplasty, kinematic alignment (KA) is proposed as an alternative to MA to minimize these issues. KA has shown equivalent or better functional outcomes and survival rates in the mid-term than MA (14). The logical next step is to apply the principles of KA in revision surgeries. The goal of KA is to restore or preserve the patient's pre-arthritic knee anatomy by resurfacing the native joint and maintaining the soft tissue envelope (11). As in primary TKA, KA principles could be applied to revision TKA. However, multiple challenges intrinsic to revision surgeries and the MA technique will need to be addressed: bone loss, loss of some anatomical landmarks, soft tissue management, revision implants, and instruments designed for MA.

**Figure 1:**
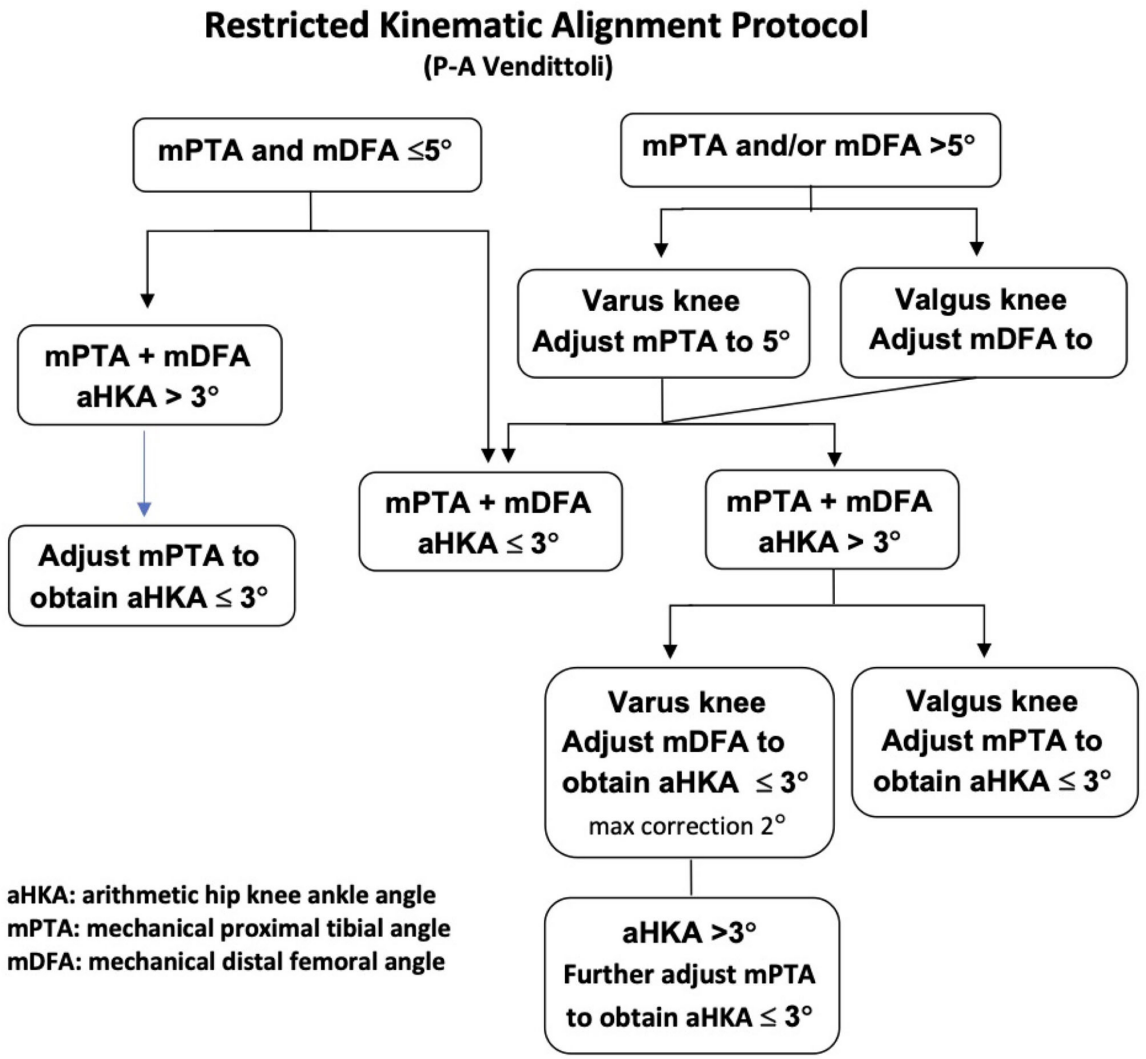
Vendittoli's restricted kinematic alignment protocol.

The senior author (PAV) has used restricted KA (rKA, Figure 1) since 2011 when performing primary TKAs, and started using rKA for TKA revision in February 2013. The study objective is to describe the technique used to perform TKA revision using rKA principles and to report early outcomes, including (1) the rerevision rate, (2) patient-reported outcome measures (PROMs) assessed with WOMAC score and, (3) radiological signs of implant dysfunction. The hypotheses for the study were that revision TKA with the use of the rKA protocol would produce favorable outcomes in PROMs, while achieving low (<10%) rerevision rates at early follow-up.

## Materials and Methods

### Patients

All revision TKAs performed by the senior author between February 2013 and November 2020 were retrospectively reviewed for inclusion in this study (*N* = 85). The following cases were excluded: revision surgeries for polyethylene liner exchange (4), for patellar resurfacing (2), with associated extra-articular deformities (1), that required long uncemented diaphyseal fixation (major metaphyseal bone loss) (15), that required hinged components (5), and 13 patients who did not want to participate in the study. A total of 42 patients (43 TKAs, one bilateral) gave their consent to participate in the study. There were 12 males and 31 females who had a mean age at the primary surgery of 67.7 years (55–85, ±7.2). Our scientific and ethics review committees approved the study, and all patients consented to include their case.

### Methods of Assessment

A retrospective review of patients' charts was used to record any rerevisions and adverse events during the follow-up period. Adverse events were recorded using the Knee Society standardized TKA complication list (16). At the last follow-up, a single research assistant assessed patients' functional outcomes using the WOMAC score (15). The post-revision anteroposterior and lateral radiographs obtained during follow-up visits were evaluated following the modern Knee Society Radiographic Evaluation System (16) to assess radiolucent lines, osteolysis, and signs of component loosening. Radiographic pre- and post-revision coronal orientation measurements were calculated in AP standing long leg x-rays using the mechanical distal femoral angle (mDFA), the mechanical proximal tibial angle (mPTA), and the arithmetic hip-knee-ankle angle (aHKA = mDFA + mPTA). Using digitized image and measurement tools, the same evaluator (LK) performed all measurements. The preoperative rotational alignment of the femoral and tibial components were measured with the use of epicondylar axis and tibial tubercle as references on CT scans as described by Berger and Crossett (17).

### Statistical Analysis

Continuous data are presented using mean, minimum, maximum, and standard deviation. Comparisons of the preoperative and postoperative continuous data were analyzed using a paired Wilcoxon test (pre- and post-revision mDFA, mPTA, aHKA). Categorial variables are presented as numbers and percentages. A significance level of *p* = 0.05 (two-sided) was used for all tests. The analyses were performed using the SPSS software version 26 (SPSS Inc., Chicago, IL, USA). No sample size calculation was done for this study as it is a cohort report with no comparison group.

### Prosthesis

The implant used in all patients for this study was the Triathlon TS Knee System (Stryker Orthopedics, Mahwah, NJ). This prosthesis is a revision system that features a single radius femoral articulating design. Tapered uncemented or cemented femoral and tibial stems are available and come in 50-, 100-, and 150-mm lengths. The stems have a fixed angle of 6° of valgus to the femoral component and neutral alignment to the tibial component. 360° of stem offset of 2–8 mm for both the femur and the tibia are available.

### Analysis of Failure to Plan rKA Reconstruction

Patients' preoperative clinical and radiographic assessments are crucial for determining the cause of failure, especially for patients with well-fixed implants, and planning the rKA reconstruction. Knees were examined for range of motion limitations and ligamentous instability. Implant size, position, orientation, and joint-line were compared with preoperative radiographic images when available or with the contralateral side. Computed tomography scans were performed to evaluate the axial rotation of the components when malrotation was suspected (17, 18). The coronal alignment was planned preoperatively using the restricted rKA protocol (Figure 1). As TKA revision systems were designed for MA with fixed coronal implant-stem angle (6° valgus on the femur and 0° on the tibia for most systems), stem relation to the meta-diaphyseal region is templated.

**Figure 2:**
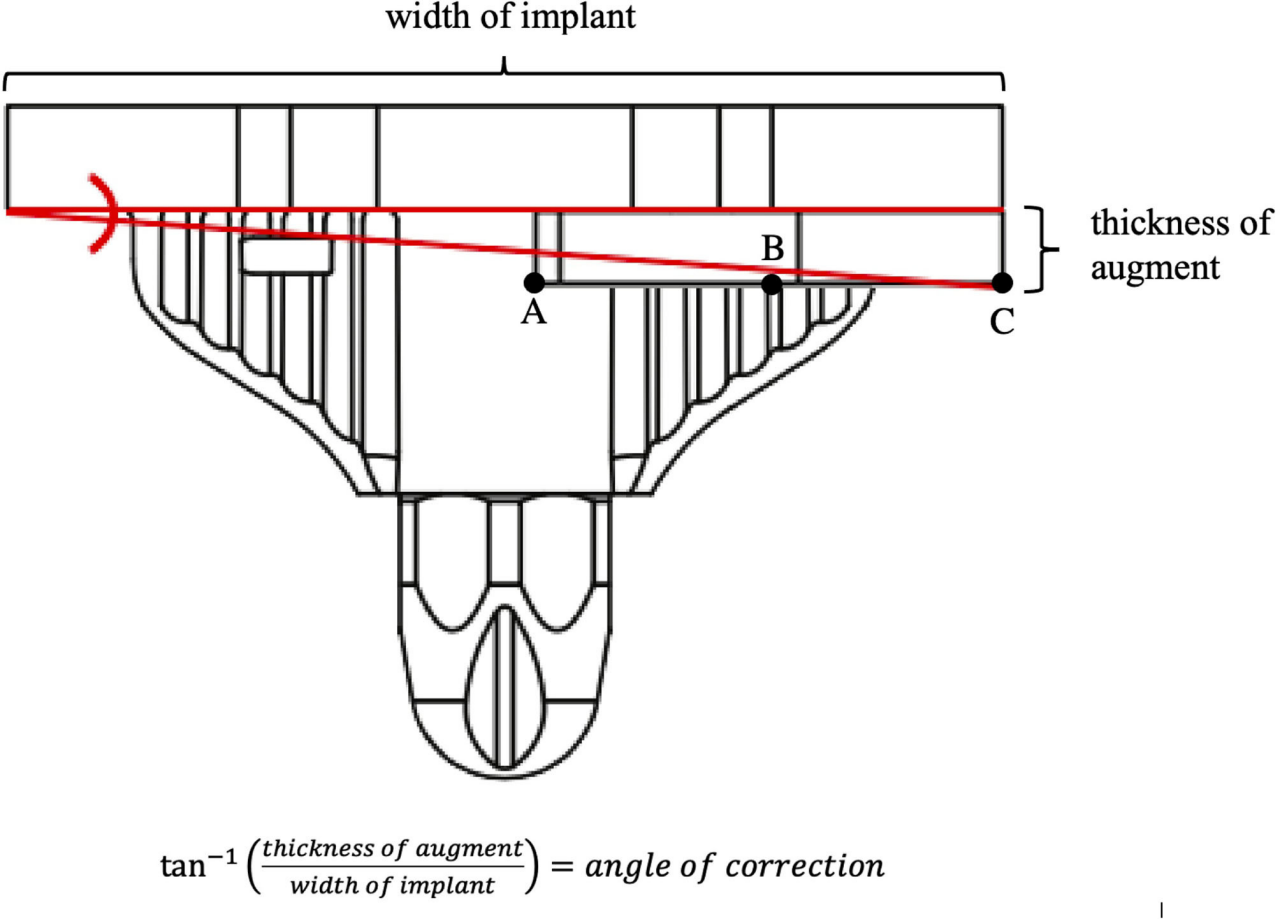
The inverse tangent of the augment's thickness over the implant's width can be used to approximate the angular correction given by apposing the component with a unilateral augment to a uniformly flat bone surface. In a purely mathematical sense, the augment would first contact a flat surface at point A. However, from a practical point of view, because of the use of cement and partial impaction in cancellous bone, using point A to calculate the angular correction would overestimate the correction. Thus point B, in the middle of the augment, was assumed to be where a flat plane would intersect the augment. The length of the segment BC was subtracted from the width of the implant in the calculations.

In most cases, to achieve the proper rKA coronal alignment, short-cemented stems are planned without interfering with the meta-diaphyseal cortex. Distal augments or bone resections needed to achieve the correct coronal angle for components was estimated. The amount of angulation achieved depends on the thickness of the augment and the size of the component (Figure 2). The approximate angular correction at the tibia for the 5- and 10-mm augments for each tibial component size are provided in Table 1. The approximate angular correction at the distal femur for the 5-, 10-, and 15-mm augments for each femoral component size are provided in Table 2. Posterior augments were utilized in a similar fashion to achieve the desired rotational alignment for the femoral component. The resulting rotational angular corrections are the same as the coronal angular corrections for the distal femoral augments already described in Table 2. If less angular correction is required, the selected augment will be combined intra-operatively with a bone resection (1–3 mm). Table 3 summarizes common problems encountered with failed primary arthroplasties and how to address them during rKA revision.

**Table 1:** Estimated angular correction given by apposing a tibial component with a unilateral augment to a uniformly flat bone surface.

**Tibia size #**	**Tibia width (mm)**	**Augment thickness (mm)**	**Angular correction (degrees)**
1	61	5	5.8
2	64	5	5.5
3	67	5	5.3
4	70	5	5.1
5	74	5	4.8
6	77	5	4.6
7	80	5	4.4
8	85	5	4.2
1	61	10	11.5
2	64	10	11.0
3	67	10	10.5
4	70	10	10.0
5	74	10	9.5
6	77	10	9.1
7	80	10	8.8
8	85	10	8.3

**Table 2:** Estimated angular correction given by apposing a femoral component with a unilateral augment to a uniformly flat bone surface.

**Femur size #**	**Femur width (mm)**	**Augment thickness (mm)**	**Angular correction (degrees)**
1	59	5	5.8
2	62	5	5.5
3	65	5	5.3
4	68	5	5.1
5	71	5	4.8
6	74	5	4.6
7	77	5	4.5
8	80	5	4.3
1	59	10	11.5
2	62	10	11.0
3	65	10	10.5
4	68	10	10.0
5	71	10	9.6
6	74	10	9.2
7	77	10	8.9
8	80	10	8.5
1	59	15	17.0
2	62	15	16.2
3	65	15	15.5
4	68	15	14.9
5	71	15	14.2
6	74	15	13.7
7	77	15	13.2
8	80	15	12.7

*Distal augments are available in 5, 10, and 15 mm sizes. Posterior augments are available in 5 and 10 mm sizes only*.

**Table 3:** Encountered problems specific to mechanically aligned primary arthroplasties revised with the rKA protocol and their solution.

**Problem**	**Diagnosis**	**Plausible solution**
Coronal malalignment of the femoral component	1) AP standing long leg x-ray 2) Preoperative x-rays 3) Contralateral knee anatomy	1) Medial distal femoral augment to correct excessive varisation from native anatomy common in MA. 2) Lateral distal femoral augment to correct excessive valgisation from native anatomy
Coronal malalignment of the tibial component	1) AP standing long leg x-ray 2) Preoperative x-rays 3) Contralateral knee anatomy	1) Lateral tibial augment to correct excessive valgisation from native anatomy common in MA 2) Medial tibial augment to correct excessive varisation from native anatomy.
Femoral axial malalignment	1) CT-Scan of the TKA	1) Posterior medial femoral augment to correct systematic external rotation in MA 2) Posterior lateral augment to correct excessive internal rotation from native anatomy.
Anterior overstuffing of the femoral component	1) Lateral x-ray of the knee 2) CT-Scan of the TKA	1) Medial and lateral posterior augments to posteriorize the femur 2) Larger medial femoral augment than lateral to correct excessive external rotation

### Surgical Technique to Apply rKA in the Revision Setting

The rKA TKA revision aims to recreate the pre-arthritic native knee anatomy and soft tissues laxities. To confirm or optimize our preoperative plan, at surgical exposure, a careful knee examination is performed to assess soft tissues laxity, knee range of motion, and the position, orientation, and fixation of the implant. After removing the implant and bone loss assessment, we used a distal femoral cutting jig connected to an intramedullary rod kept loose in the metaphysis (not deeply inserted, to avoid a tight fit in the diaphysis). We performed the distal femoral refreshing cuts including the planned supplemental bone resection and/or metallic augments to modify the mDFA adjust the joint line level when required (Table 2). In practice, a 5-mm augment angulates the component by 5°. When dealing with the smallest sizes, this angle would be closer to 6° and 4° with the largest components. Using an anterior referenced 4-in-1 femoral cutting block of the appropriate size, and positioned to correct any malrotation, we performed the anterior, chamfer, posterior, and posterior stabilized (PS) box cuts. Then, we performed the proximal tibial refreshing cuts using a cutting jig connected to an intramedullary rod kept loose in the metaphysis. Tibial cut orientation included the planned supplemental bone resection and/or metallic augments to modify the mPTA when required while keeping the tibial slope neutral (Table 1).

With trial implants in place, the collateral ligaments' laxities in 10° of flexion are assessed to determine the polyethylene thickness and serve as an indicator for achieving a planned coronal alignment (goal is 1–2 mm of medial and 2–3 mm of lateral joint opening). Next, the medial and lateral flexion gaps are assessed (goal is 2–3 mm of medial and 3–4 mm of lateral joint opening). If present, hyperlaxity is addressed by increasing the femoral size, using posterior augments. In cases of significant mediolateral imbalances (>4 mm difference) or gross flexion instability with the larger femoral component compatible with the selected tibial size, a varus-valgus constrained liner (TS) was used. Such imbalances were present in cases with longstanding soft tissue changes: ligament stretching or contractures where complete capsulectomy and debridement was required to obtain satisfactory ROM. Lastly, the patella was left intact if well tracking or revised if resurfaced with malposition or under resection.

## Results

Indications for performing knee revision are presented in Table 4. All interventions included both femoral and tibial components revision, except for one case where only the femoral component was revised for aseptic loosening. Cemented stems were used for both the femur and tibia in 28 (65%) cases, for the femur alone in 13 (30%) cases, and no stems in two cases. The stem lengths are presented in Table 5. Thirty-nine (91%) cases required femoral side augments, one case required augments in both femoral and tibial components, and three cases required no augments. A PS tibial insert was used in 31 (72%) cases, while 12 (28%) cases required a more constrained TS insert. The patella was resurfaced during the primary surgery in 39 (91%) cases, kept as is in 20 (47%) cases, and revised in 19 (44%) cases. Four (9%) patella were not resurfaced in any surgery. Mean surgical time was 102 min (66–156, ±18). Mean intraoperative blood loss was 236 ml (50–600, ±121).

**Table 4:** Indications for revision (there were revisions with more than one factor leading to revision indication).

**Indication**	***N***
**Aseptic component loosening**	
Femoral component	7
Tibial component	6
Both components	–
**Component malpositioning**	
Femoral component	
Coronal	Valgus: 3 Varus: 7
Axial	Internal rotation: 4 External rotation: 1
Sagittal	Flexion: 1
Tibial component	
Coronal	Valgus: 2 Varus: 3
Axial	Internal rotation: 2 External rotation: 10
Sagittal	Flexion: 4
**Anterior overstuffing**	
Femoral component anteriorized	2
Patella under resection	2
**Sizing error**	
Femoral component	Oversized: 3; undersized: 2
Tibial component	Oversized: 1; undersized: 1
**Joint line**	Distalized: 3 Proximalized: 1
**Osteolysis**	
Femoral side	2
Tibial side	2
Both sides	4
**Pain as one of the main factors**	23
**Ligamentous instability**	10
**Polyethylene wear**	2
**Septic failure**	3
**Patellar instability**	2
**Stiffness**	12

**Table 5:** Stem length details.

**Stem length**	**Femur** ***N*** **(%)**	**Tibial** ***N*** **(%)**
No stem	3 (7%)	5 (11.6%)
Stubby/bullet tip	0	12 (27.9%)
50 mm	35 (81.4%)	25 (58.1%)
100 mm	5 (11.6%)	1 (2.3%)

After a mean post-revision follow-up of 4 years (0.9–7.7, ±2), no patient was lost to follow-up, and there was only one case of reoperation. This case is a female aged 67 years at the time of her index primary TKA in 2012. We performed a revision surgery in 2014 for instability. After the revision, she complained of persisting femorotibial instability. In 2016, we performed a simple polyethylene exchange from a PS to a TS insert. At the final follow-up at 78 months, this patient had a WOMAC score of 61 and a ROM of 0–130°.

There was a total of four adverse events requiring conservative treatment. First, there were two postoperative periprosthetic fractures due to trauma (one undisplaced metaphyseal femoral fracture and one transverse patellar fracture) treated conservatively and healed uneventfully. One patient developed ipsilateral thromboembolic disease and was treated with anticoagulants. Finally, one patient developed a wound complication (localized superficial wound infection) and was treated with antibiotics alone.

At the last follow-up, the mean WOMAC score was 34.4 (0–80, ±21.7). There were 14 (32.6%) patients who complained of persisting knee pain despite knee revision. These patients had a mean WOMAC score of 36.5 (3–71, ±20.5). In most cases, the pain level was reported as improved compared to the pre-revision level and could not be attributed to any specific cause. Two of these patients experienced mild pain associated with unresolved relapsing knee effusion. In one patient, the pain was attributed to a painful neuroma of the infrapatellar branch of the saphenous nerve. Finally, in one patient, the pain was thought to be of neuropathic origin, and the patient was referred to the pain clinic for further treatment.

There were no radiolucencies or osteolysis noted on radiographic evaluation. Pre- and post-revision radiographic measurements are provided in Table 6.

**Table 6:** Radiographic measurements (negative value represents varus and positive represents valgus).

**Radiographic measurements**	**Pre-revision**	**Post-revision**	***P*** **-value**
aHKA mean (min–max, ±SD)	−1.8 (−19–7, ±4.4)	−0.8 (−5–4, ±2.1)	0.172
mDFA mean (min–max, ±SD)	0.4 (−8–8, ±3.2)	1.7 (−2–5, ±1.6)	0.678
mPTA mean (min–max, ±SD)	−2.2 (−11–2, ±2.4)	−2.5 (−5–1, ±1.4)	0.518

*aHKA, arithmetic mechanical hip-knee-ankle angle (mDFA + mPTA); mDFA, mechanical distal femoral angle; mPTA, mechanical proximal tibial angle; SD, standard deviation*.

### Case Examples

To illustrate the type of cases included in the present cohort, three cases examples are presented.

#### Case 1

**Figure 3:**
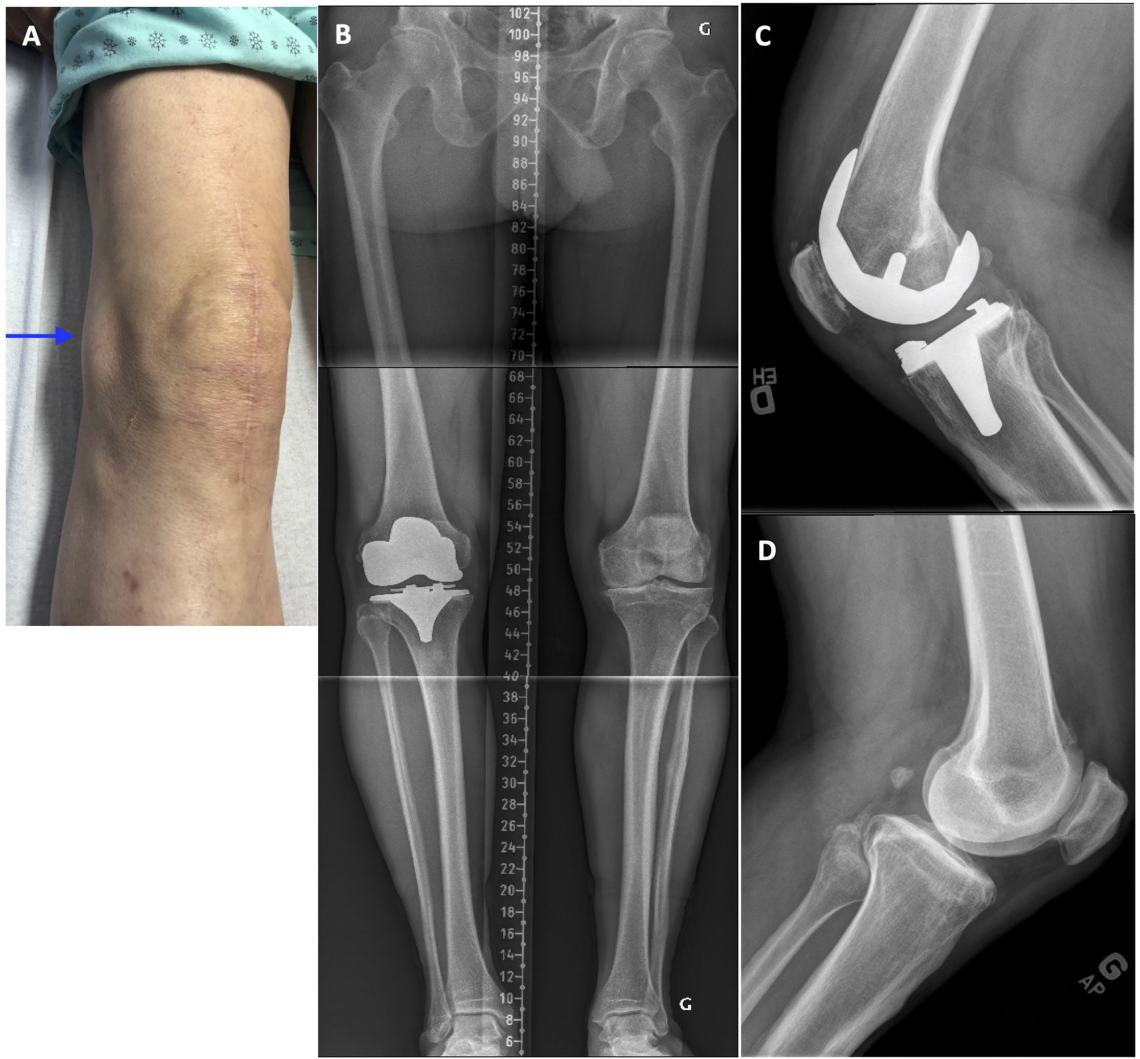
**(A)** Right knee clinical examination where swelling of the biceps tendon is observed. **(B)** Pre-revision long leg standing X-Ray reveals a right TKA implant with mDFA of 4° varus and a mPTA of 1.5° varus (5.5° varus aHKA). On the intact left side, the mDFA is 3.0° valgus and mPTA 3.5° varus (aHKA of 0.5° varus). **(C)** right knee lateral view where the implant posterior tibial slope is 6.5°. **(D)** Left knee lateral view where the native tibial slope is 2.0° posterior.

**Figure 4:**
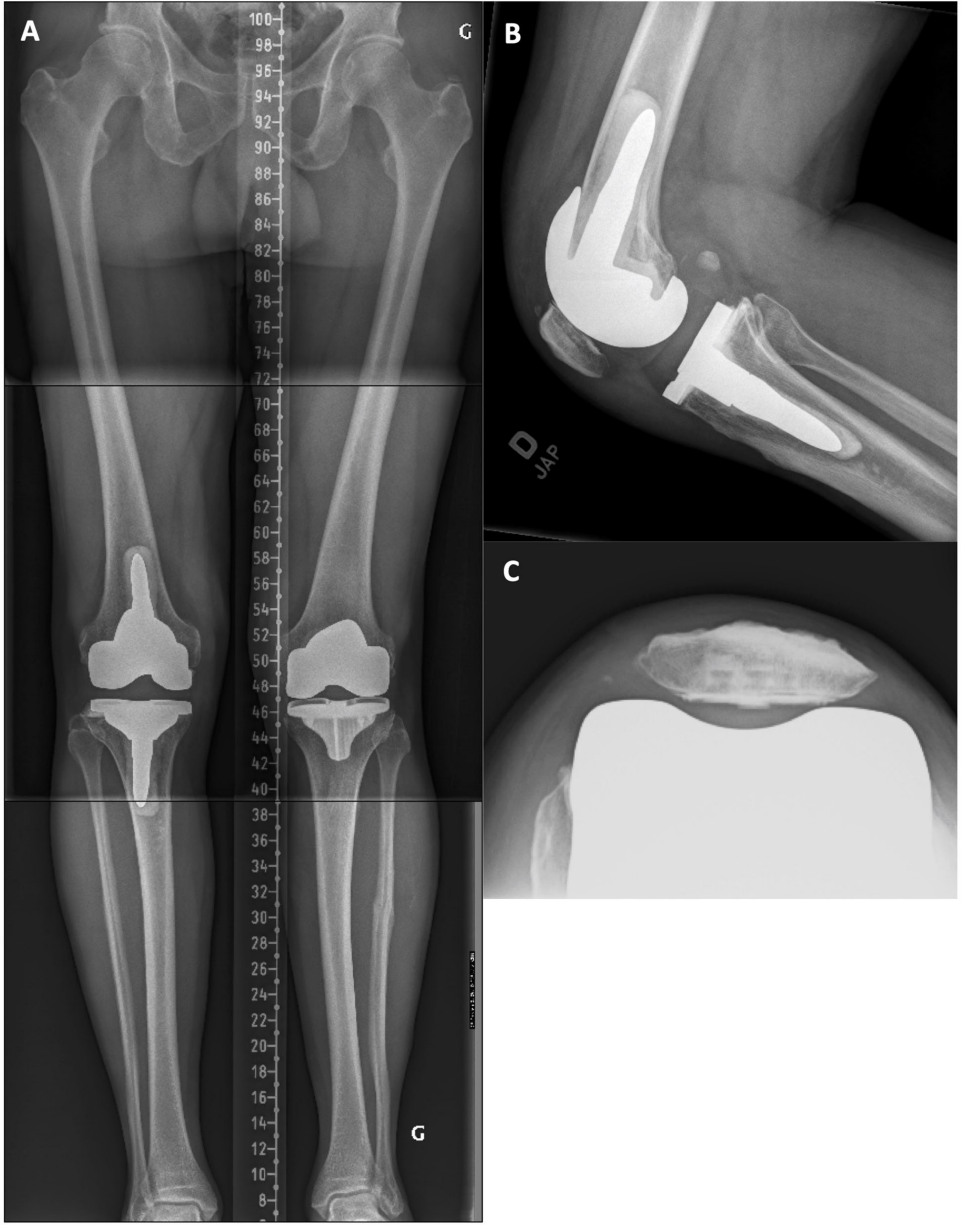
**(A)** Post-revision standing long-leg X-ray of the right lower limb where the implant mDFA has been modified to 3.0° valgus and the mPTA at 4.0° varus (1.0° varus aHKA). As expected, the 50 mm cemented stems are not aligned with the tibial and femoral bone diaphysis and comes in contact with the lateral cortex on the tibial side. **(B)** Right knee lateral view where the implant posterior tibial slope has been corrected to 2.0°. **(C)** Right patella skyline view showing a well-centered implant.

A 61-year-old male with a painful and unstable right TKA 2 years after the surgery. At clinical examination, important MCL laxity was observed along with a biceps femoris tendinitis (see Figure 3A and Supplementary Video 1). Compared to the intact contralateral lower limb, the prosthetic knee was implanted with increased femoral varus (+7.0°), decreased tibial varus (–2.0°), and increased posterior tibial slope (+4.5°, Figures 3B–D). During revision surgery and after implant removal (no bone loss), to correct the mDFA by 7°, a 5-mm distal medial femoral augment was used in combination with a lateral distal condyle bone resection of 2 mm. Tibial bone surface was refreshed, by removing 4 mm of anterior bone (none posteriorly, reducing the slope) and 2 mm medially to adjust varus/valgus orientation. With trial implants in place, the observed laxities (MCL 1–2 mm and LCL 3–4 mm) at 10° of flexion confirmed that we achieved our goals. As diaphyseal stem fixation would prevent the restoration of the patient's joint orientations, on both femoral and tibial sides, 12 × 50-mm cemented stems were used. A standard PS insert was selected (Figures 4A–C). At 18 months follow-up, the patient reported to be pain-free with a WOMAC score of 15 and a ROM of 0–125°.

#### Case 2

**Figure 5:**
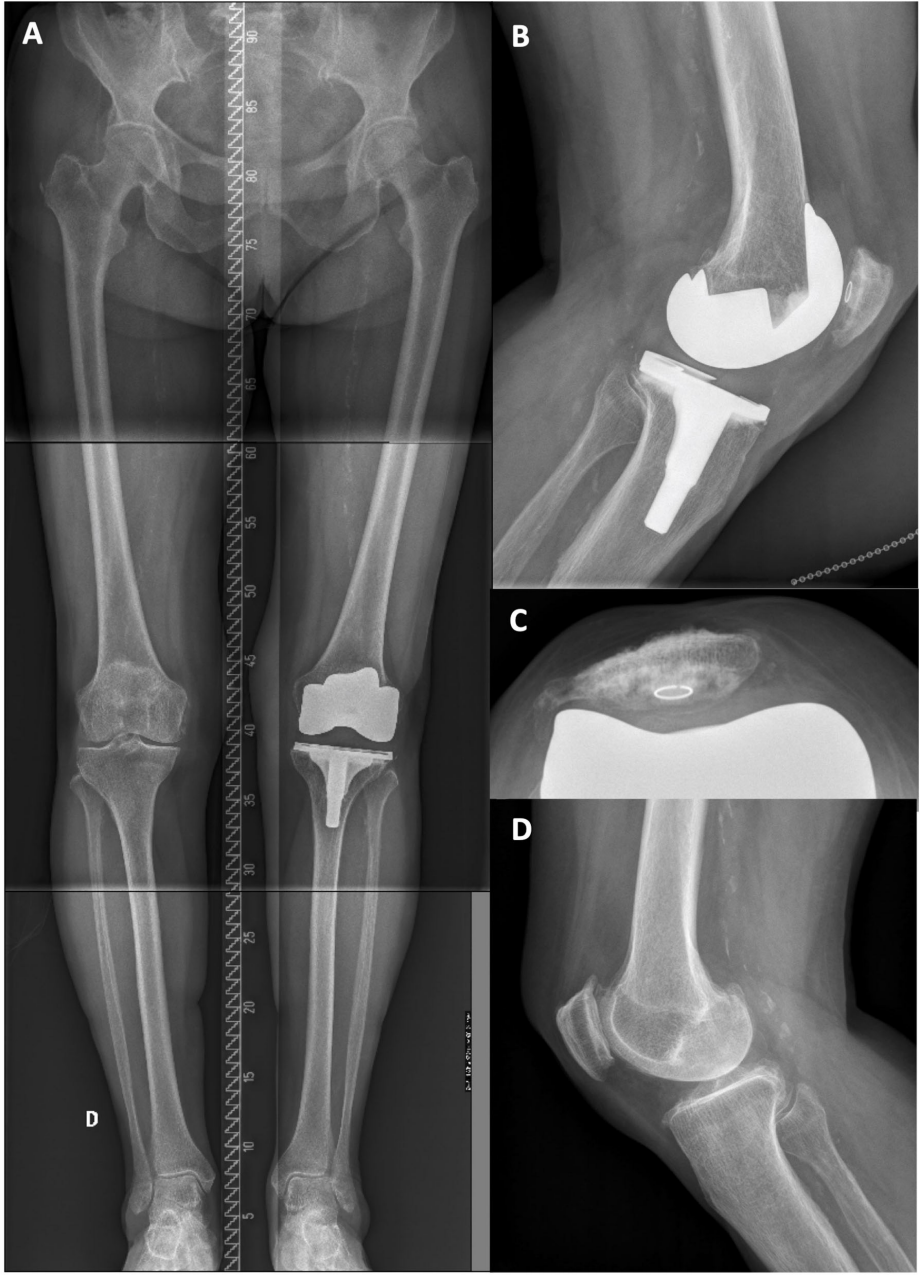
**(A)** Pre-revision long leg standing X-Ray reveals a left implant mDFA of 2° varus and an mPTA of 2° valgus (0° varus aHKA). On the intact right side, the mDFA is 2.0° valgus and mPTA 3.5° varus (aHKA of 1.5° varus). **(B)** Left knee lateral view where the implant posterior tibial slope is 12.0°. **(C)** Left patella skyline view showing a lateral patellar retinaculum calcification and a well-centered resurfaced patella. **(D)** Right knee lateral view where the native tibial slope is 6.0° posterior.

**Figure 6:**
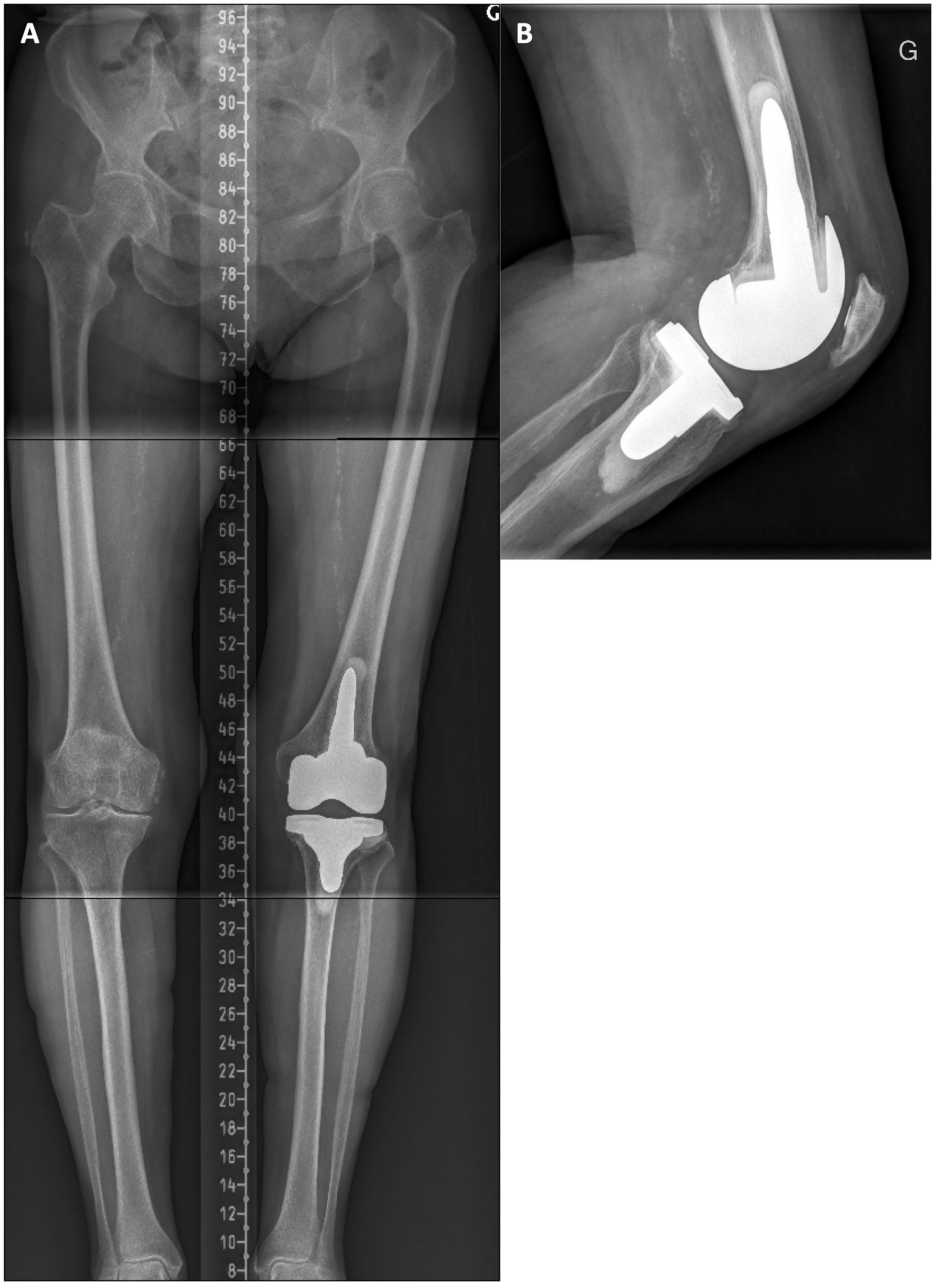
**(A)** Post-revision standing long-leg X-ray of the left lower limb where the implant mDFA has been modified to 2.0° valgus and the mPTA at 3.0° varus (1.0° varus aHKA). As expected, the femoral cemented stems is not aligned with the femoral bone diaphysis. On the tibial side, to restore patient's alignment, only a stubby stem could be used and comes in contact with the lateral cortex. **(B)** Left knee lateral view where the implant posterior tibial slope has been reduced to 3.0°. Because manufacturer recommendations for this implant is a neutral slope, we did not aim at patient's contra-lateral side value of 6°.

A 75-year-old female was unsatisfied with the clinical results of her left TKA, 5 years after the surgery. There was a mid-flexion instability at clinical examination with a medial opening of 5 mm at 45° of flexion and a total ROM of 0–90°. On radiographic examination (Figures 5A–D), there was an oversized tibia (lateral overhang), a lateral patellar retinaculum calcification, and, compared to the intact contralateral lower limb, the prosthetic knee was implanted with increased femoral varus (+4.0°), decreased tibial varus (–5.5°), and increased tibial posterior slope (+6°). During revision surgery and after implant removal, to correct the mDFA by 5° and lower the elevated joint line, a 10-mm distal medial femoral augment was used in combination with a 5-mm augment on the lateral side after a 2-mm refreshing cut. Posterior augments (5 mm) were used medially and laterally. The tibial bone surface was cut by removing 5 mm of anterior bone (none posteriorly, reducing the slope) and 5 mm medially to adjust varus/valgus orientation. With trial implants in place, the observed laxities (MCL 2 mm and LCL 3 mm) at 10° of flexion confirmed that we achieved our goals. With the patient's bone anatomy, a 50 × 12-mm femoral stem and a short tibial stubby were cemented. A standard PS insert (13 mm) was selected (Figures 6A,B). At 53 months follow-up, the patient reported no pain and significantly improved with a WOMAC score of 13 and a ROM of 0–115°.

#### Case 3

**Figure 7:**
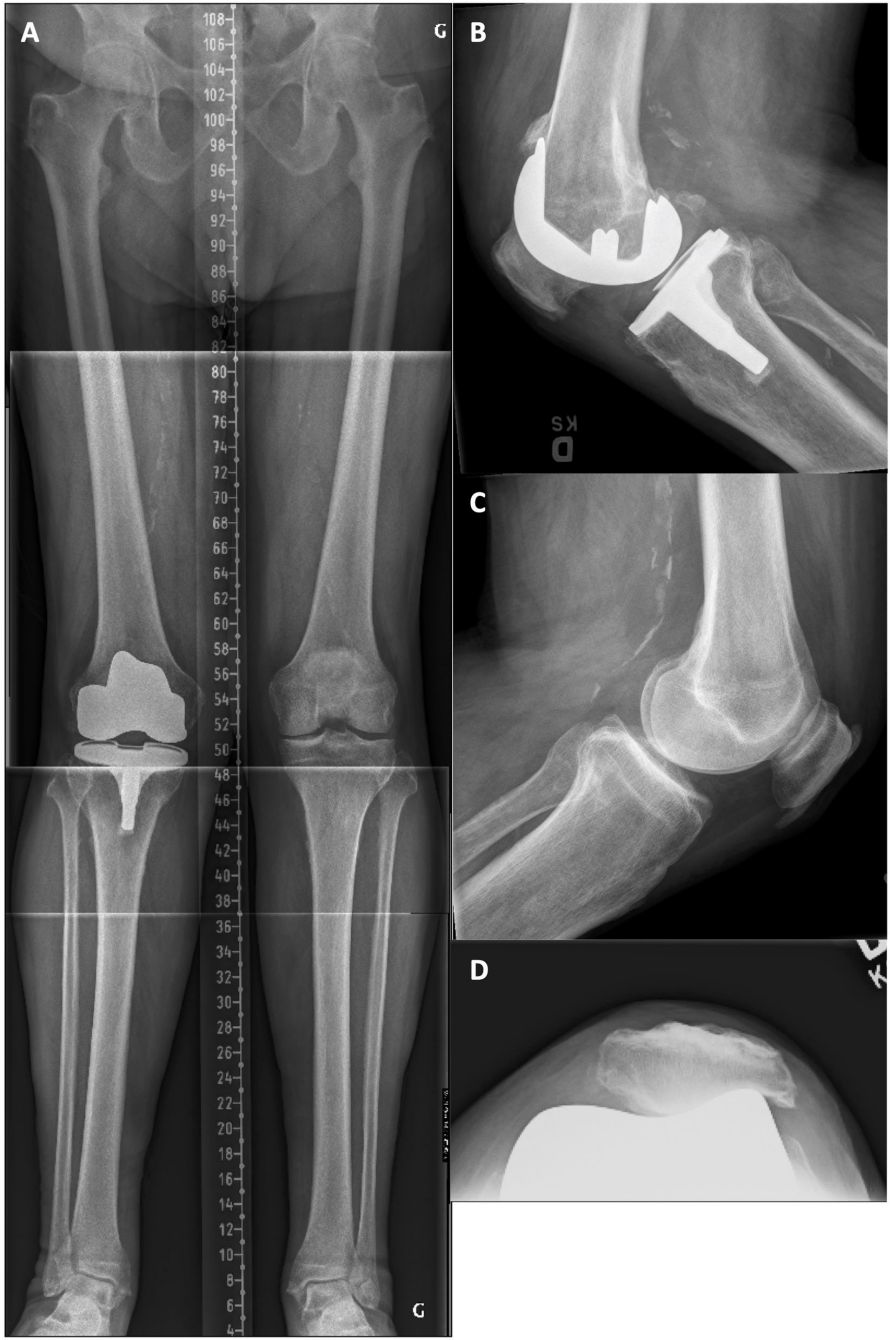
**(A)** Pre-revision long leg standing X-Ray reveals a right implant mDFA of 4° valgus and an mPTA of 1.5° valgus (5.5° valgus aHKA). On the intact left side, the mDFA is 2.0° valgus and mPTA 0.5° varus (aHKA of 1.5° valgus). **(B)** Right knee lateral view where the implant tibial slope is 1.5° anterior. **(C)** Left knee lateral view where the native tibial slope is 8.0° posterior. **(D)** Right knee skyline view showing a subluxed and worn patella.

**Figure 8:**
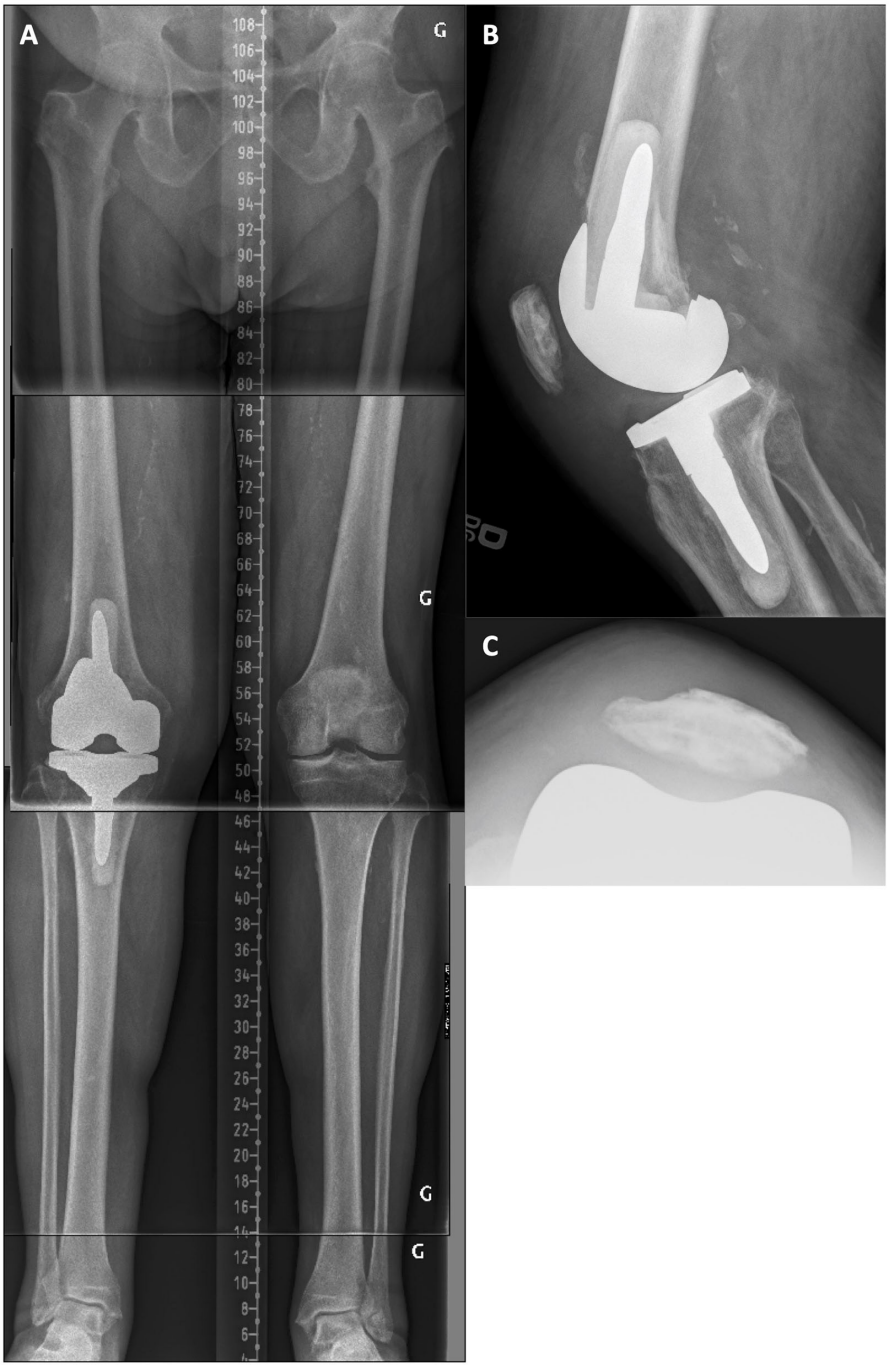
**(A)** Post-revision standing long-leg X-ray of the right lower limb where the implant mDFA has been modified to 1.0° valgus and the mPTA at 0.5° varus (0.5° valgus aHKA). **(B)** Right knee lateral view where the implant tibial slope has been shifted from 1.5° anterior to 2.0° posterior. The femoral implant has also been translated posteriorly to be flush with the anterior cortex. **(C)** Right knee skyline view showing a well-centered resurfaced patella.

A 76-year-old male with a painful left TKA 4 years after the surgery. At clinical examination, the knee had a flexion contracture of 15° and reached 90° of flexion. There was profound medial femoral and patellar pain upon palpation. On radiographic examination, compared to the intact contralateral lower limb, the right operated side was implanted with increased femoral valgus (+2°), decreased tibial varus (−2.0°), and reduced tibial posterior slope (−9.5°, Figures 7A–D). In addition, the unresurfaced patella was subluxed and severely worn. Posterior femoral offset was estimated to be 5–7 mm shorter, and the femoral implant translated anteriorly. After implant removal, in addition to a refreshing cut, a supplemental 2-mm resection on the distal lateral condyle was performed to correct the mDFA by 2°. To maintain the joint line, 5-mm distal augments were used on both condyles. To compensate for the posterior femoral condyles' asymmetry of the implant in place (Genesis femur from Smith and Nephew has a thicker medial condyle), and to increase posterior femoral offset, we used a 10-mm posterior augment on the medial side and a 5-mm augment on the lateral side. Tibial bone surface was cut by removing 5 mm of anterior bone (none posteriorly, reducing the slope) and 2 mm medially to adjust varus/valgus orientation. With trial implants in place, the MCL laxity was 5 mm larger than LCL at 10° of flexion. This difference was hypothesized to be secondary to long-term tension on the MCL and subsequent stretching. In such a situation, we preferred to use a semi-constrained implant (Figures 8A–C). At 16 months follow-up, the patient reported having minimal pain and a ROM of 0–125°.

## Discussion

The most important finding of the present study was that rKA principles can be safely used in revision TKA in the short- to mid-term, thus supporting our hypothesis. At a mean 4 years' follow-up, only one (2.3%) subject in our study required a reoperation for a polyethylene exchange.

Most revision TKA cases included in this study had unsuccessful clinical results of the primary joint replacement (persisting pain, stiffness, instability, etc.). To improve patients' function and satisfaction, the rKA protocol was used for revision TKA. In contrast to the studies listed in Table 7 where TS inserts were used almost systematically, the rKA protocol allowed most of our cases to be balanced with a standard PS insert (72%) and we obtained one of the lowest reoperation rates.

**Table 7:** Results of studies reporting their outcomes with the Triathlon TS Knee System (Triathlon Total Knee System; Stryker Orthopedics, Mahwah, NJ) for revision TKA.

**References**	**N TKAs/** **mean FU in years**	**Indication for revision**	**Type of PE insert**	**Component fixation**	**Rerevisions**	**PROMs** **preoperatively mean** **(range, ±SD)**	**PROMs** **at final follow-up mean** **(range, ±SD)**	**Radiological analysis**
Gwam et al. (19)	93/4	NR, septic failures were excluded	TS	NR	4 aseptic loosening 2 septic failures	NR	KSS: 86 (38–100) Functional KSS: 52 (15–90)	Excluding the revised cases, there were no progressive radiolucencies or osteolysis noted
Hamilton et al. (10)	53/2	NR, septic failures were excluded	TS	Fully cemented	None	OKS: 19.1 (±7.41)	OKS: 36.4 (±8.2)	NR
Stevens et al. (20)	100/7.4	85 aseptic reasons 15 septic reasons	TS	Fully cemented	6 septic failures 3 instability 1 infected periprosthetic fracture 2 reoperations for additional patellar resurfacing	NR	OKS: 27 (0–46, ±11.9) FJS: 32.3 (0–100, 30.4) SF-12 PCS: 40.6 (23.9–67.1 ±17.6) SF-12 MCS: 48.3 (23.9–69.1, ±15.5)	Excluding the revised cases, nine postoperative radiographs demonstrated non-progressive radiographic lucent lines with no evidence of loosening. One radiograph demonstrated progression of radiographic lucent lines and lysis
Limberg et al. (21)	416/4	122 instability 105 aseptic loosening 97 septic failure 92 other	TS	Fully cemented	23 septic failures 17 instability 10 aseptic loosening 1 arthrofibrosis 1 periprosthetic fracture 1 patella fracture	KSS: 46	KSS: 81	NR
Present study (2021)	43/4	40 aseptic reasons 3 septic reasons	31 PS 12 TS	Fully cemented	1 instability	NR	WOMAC: 34.4 (0–80, ±21.7)	No progressive radiolucencies or osteolysis noted

*KSS, Knee Society Score; OKS, Oxford Knee Score; FJS, Functional Joint Score; SF-12 PCS, Physical Component Summary; SF-12 MCS, Mental Component Summary; SD, standard deviation; NR, Not reported*.

The PROMs of revision knee arthroplasty using rKA in our study showed a WOMAC score of 34.4 (0–80, ±21.7) at the last follow-up. We also found that 14 (33%) patients reported persisting anterior knee pain after their revision surgery, even though we did not use a pain scale to quantify this finding. PROMs and satisfaction rates differ between primary and revision TKAs, with revision surgeries showing less improvement (4, 22). It is very difficult to evaluate the functional results of patients with revision TKA because of the high heterogeneity of the causes of failure. The primary operation has a substantial influence on the postoperative outcome of the revision. Baker et al. (4), analyzing data from the National Joint Registry for England and Wales (NJR) found the highest improvements in PROMs and satisfaction in cases where aseptic loosening was the cause and the lowest improvements when stiffness was the cause for revision. Greidanus et al. (22) studied 60 TKA revision surgeries and found a total WOMAC score of 30.9 at 2 years after surgery. Kasmire et al. (23) followed 175 patients who underwent revision TKA for aseptic failure and reported a total WOMAC score of 28.1 at 2 years' follow-up. Notably, there is a paucity of literature regarding WOMAC scores for revision TKA at more than 2 years, and even though our scores do not show a superiority over the MA technique, we can assume that our WOMAC results would be at least comparable to projecting the results of the previous studies to a longer follow-up.

Radiographic analysis in the present study showed no radiolucencies or signs of loose components. Therefore, standing coronal alignment was changed from 1.8° to 0.8° of aHKA varus to recreate the native knee anatomy set within the limits of rKA protocol. Our findings suggest that rKA used in revision TKA does not preclude good outcomes for revision arthroplasty.

This study and the proposed rKA technique for revision TKA are not without limitations. First, to be eligible for a revision with the rKA protocol, a patient must be eligible to be revised with a short-cemented stem. If a longer stem with diaphyseal fixation is required, the longer stem may not be compatible with the patient's anatomy and its restoration. Therefore, it is plausible that some of the more complicated revision cases with severe bone loss or instability requiring hinge implant, for example (22/85 cases), were excluded from this study. This might explain our lower rerevision rate compared to other studies. Second, our study is limited by its small sample size (43 revision TKAs carried out by only one surgeon) and this might contribute to overlooking the increased risk for aseptic loosening and recurrent infection after revision surgery. However, it is a continuous series, and we believe that our cohort is representative of an academic center revision practice. Second, our study has a short mean follow-up (4 years), and longer follow-up studies are warranted to evaluate the long-term safety of this technique. Third, many patients that participated in our study were referred to us from different institutions and data regarding the primary surgery were not available to us. Therefore, because of the retrospective nature of this study, we could not measure the improvement in PROMs from primary surgery.

We believe that this study, being the first of its kind, will spark the interest in the orthopedic community to use rKA for revision TKA, especially in the cases of early, non-wear-related unsuccessful MA TKAs. It is agreed that using precision tools like navigation or robot is the future way to go to perform such procedures. Navigation in the setting of rKA revisions has been used by the authors (PAV, MOK). While this technology helps to make accurate refresh cuts, the actual design of the cutting block made for primary TKA makes it difficult to stabilize in cases with significant metaphyseal bone loss. It also does not allow the surgeon to perform step cuts. In the near future, robotic surgery may prove to be an appealing option to facilitate rKA knee revisions once the appropriate software is available.

Last, there is limited scientific evidence to define the acceptable lower limb alignment and joint line tilt limits and related implants orientations. Such evidence, supporting the universal use of KA in extreme anatomies, may allow removing rKA boundaries. Nevertheless, in the meanwhile, we believe that rKA is a safe and favorable new technique for TKA revision.

## Conclusion

Although current revision TKA implants are not ideal for revision TKA performed with rKA; it is an appealing alternative to MA in the mid-term, especially in the cases of early, non-wear-related unsuccessful MA TKAs.

## Data Availability Statement

The raw data supporting the conclusions of this article will be made available by the authors, without undue reservation.

## Ethics Statement

The studies involving human participants were reviewed and approved by Comité d'éthique de la recherche CIUSSS de l'Est-de-l'Île-de-Montréal. The patients/participants provided their written informed consent to participate in this study. All patients consent for inclusion of their case.

## Author Contributions

LK, GBR, SM, M-OK, and JB collected the data and wrote the manuscript. JB carried out the statistical analysis. P-AV was involved in the experimentation and surgery performance and performed the surgeries, designed the study, reviewed the article, and was responsible for manuscript submission. All authors have approved the final article.

## Conflict of Interest

P-AV reports grants, personal fees, and royalties from Microport Inc., grants and personal fees from Stryker, grants and personal fees from Medacta, and grants and personal fees from Johnson and Johnson, outside the submitted work. The remaining authors declare that the research was conducted in the absence of any commercial or financial relationships that could be construed as a potential conflict of interest.

## Publisher's Note

All claims expressed in this article are solely those of the authors and do not necessarily represent those of their affiliated organizations, or those of the publisher, the editors and the reviewers. Any product that may be evaluated in this article, or claim that may be made by its manufacturer, is not guaranteed or endorsed by the publisher.

## Abbreviations

aHKA: Arithmetic hip–knee–ankle angle
BMI: Body mass index
KA: Kinematic alignment
MA: Mechanical alignment
mDFA: Mechanical distal femoral angle
mPTA: Mechanical proximal tibial angle
PROMs: Patient reported outcome measures
PS: Posterior stabilized
RCT: Randomized controlled trial
rKA: Restricted kinematic alignment
TKA: Total knee arthroplasty
TS: Total stabilizer
WOMAC: Western Ontario and McMaster Universities Osteoarthritis Index.
